# Th17-Associated Cytokines as a Therapeutic Target for Steroid-Insensitive Asthma

**DOI:** 10.1155/2013/609395

**Published:** 2013-12-23

**Authors:** Yuko Morishima, Satoshi Ano, Yukio Ishii, Shigeo Ohtsuka, Masashi Matsuyama, Mio Kawaguchi, Nobuyuki Hizawa

**Affiliations:** Division of Clinical Medicine, Department of Respiratory Medicine, Faculty of Medicine, University of Tsukuba, 1-1-1 Tennoudai, Tsukuba, Ibaraki 305-8575, Japan

## Abstract

Steroid-insensitive asthma is an infrequent but problematic airway disease that presents with persistent symptoms, airflow limitation, or recurrent exacerbations even when treated with steroid-based therapies. Because of unsatisfactory results obtained from currently available therapies for steroid-insensitive asthma, a better understanding of its pathogenesis and the development of new targeted molecular therapies are warranted. Recent studies indicated that levels of interleukin (IL)-17 are increased and both eosinophils and neutrophils infiltrate the airways of severe asthmatics. IL-17 is a proinflammatory cytokine mainly secreted from helper T (Th) 17 cells and is important for the induction of neutrophil recruitment and migration at sites of inflammation. This review focuses on the pathogenetic role of Th17 cells and their associated cytokines in steroid-insensitive asthma and discusses the prospects of novel therapeutic options targeting the Th17 signaling pathway.

## 1. Introduction

Asthma is a very common disease that affects many people, men and women, young and old, worldwide. Although asthma is mostly well controlled by conventional therapies including inhaled corticosteroids, about 5–10% of asthma patients have a severe phenotype described as “fatal or near-fatal asthma,” “severe asthma,” “steroid-dependent asthma,” “steroid-insensitive asthma,” “difficult to control asthma,” “poorly controlled asthma,” “brittle asthma,” or “irreversible asthma” [1]. The causes of these conditions are complex and most likely heterogeneous. Some can be explained by insufficient or inadequate treatment, while others explained by airway inflammation that is resistant to conventional treatment. Continuous exposure to aggravating factors and/or associated comorbid conditions may exert a deleterious influence on asthma control, but a certain type of airway inflammation may also contribute to standard therapy unresponsiveness. As such, the pathogenesis of uncontrollable asthma, especially steroid-insensitive asthma, has been a long-standing interest among researchers attempting to establish a novel strategy for the treatment of patients with persistent symptoms, irreversible airflow obstruction, or frequent exacerbations even under adequate treatment.

The current consensus on asthma is that the main underlying pathology is chronic airway inflammation in which inflammatory cells, such as eosinophils and helper T (Th) 2 lymphocytes, play a role. The Th1/Th2 paradigm has offered important insights into the pathogenesis of asthma, and there is no doubt that this classical Th1/Th2 theory mostly explains the immune responses in asthma. Based on the idea that asthma is associated with polarized Th2 responses, various clinical trials have been conducted to develop effective new therapeutic options by adjusting the Th1/Th2 cytokine balance. Several studies have demonstrated the benefits of an IL-4 variant, a soluble recombinant human interleukin (IL)-4 receptor, anti-IL-5 monoclonal antibodies, and anti-IL-13 monoclonal antibodies in controlling respiratory symptoms or in preventing either bronchospasm or eosinophilic airway inflammation in asthmatic patients [2–6]. However, some studies failed to show complete improvements in therapeutic outcomes by blocking the biological actions of Th2 cytokines [7, 8], and some limitations have been recognized to exist in the Th1/Th2 paradigm.

Over the last two decades, our understanding of the pathogenic role of various Th cell subsets has greatly advanced. Several studies have recently described the characteristics of severe asthma to include the involvement of neutrophils as well as that of eosinophils [1, 9–12]. Neutrophilic airway inflammation appears to be insensitive to steroids [1, 11, 13] and may be related to Th17 rather than Th2 cytokines [14–17]. This review highlights the role of Th17 cells in the pathogenesis of steroid-insensitive asthma and discusses the possibilities of developing new therapeutic options targeting Th17 cells and their related cytokines.

## 2. General Features of Th17 Cells

### 2.1. Th17 Cell Differentiation

The network of differentiation factors and their interactions for generating Th17 cells are intricate and finely balanced, and they have gradually become understood. Th17 cells are derived from T cell precursors, naïve CD4^+^ T cells common to Th1, Th2, and regulatory T (Treg) cells [18, 19]. The differentiation of naïve CD4^+^ T cells into each cell type is triggered by a particular cytokine milieu during stimulation by cognate antigen. For Th17 cells, transforming growth factor (TGF)-*β* and IL-6, together with IL-21 and IL-23, may play a role in the induction of Th17-cell differentiation and activation [20, 21]. Since Th17 cells have a key role in the secretion of IL-21 and IL-6 produced by themselves or by stimulating other target cells, an amplification loop may exist to enhance Th17 cell differentiation through autocrine and paracrine regulation [22, 23]. Other proinflammatory cytokines such as IL-1*β* and tumor necrosis factor (TNF)-*α* also promote the development of Th17 cells [24, 25]. By contrast, several studies have demonstrated a different regulatory mechanism where TGF-*β* is not required for Th17 cell differentiation [26–28]. Nevertheless, the present consensus among researchers is that TGF-*β* is usually required for generating Th17 cells [29–31]. Upregulation and activation of the key transcription factors are also crucial for Th17 cell differentiation. Signaling transducer and activator of transcription 3 (STAT3) and retinoic acid-related orphan receptor-*γ*t (ROR*γ*t), recently described as a Th17 master regulator, are both known to be important for Th17 cell differentiation and the production of related cytokines [20, 21, 32].

Th17 cell differentiation is also controlled by other T cell lineages and their associated cytokines. The Th1 cytokine interferon (IFN)-*γ* and Th2 cytokine IL-4 exert negative effects on the differentiation of Th17 cells [18, 19], while IL-9 exerts a promoting effect [33]. T-bet and Foxp3, master transcription factors for Th1 and Treg cell differentiation, respectively, may negatively regulate Th17 cell differentiation by interacting with ROR*γ*t to suppress its function [34, 35]. Interestingly, the multifunctional cytokine TGF-*β*, produced by every leukocyte lineage and also by nonimmune cells, has pleiotropic properties on the differentiation of Th17 and Treg cells. TGF-*β* displays distinct functions depending on the presence of IL-6. TGF-*β* alone leads to the differentiation of Treg cells that have suppressive effects on excessive Th1/Th2/Th17 immune responses, whereas TGF-*β*, together with IL-6, promotes the differentiation of Th17 cells. In addition, it was demonstrated that not only the combination but also the amount of cytokine stimuli is a critical determinant for T cell differentiation. Low concentration of TGF-*β* enhances the generation of Th17 cells, whereas high concentration promotes the development of Treg cells and inhibit that of Th17 cells [35].

### 2.2. Signature Cytokines of Th17 Cells, IL-17A, and IL-17F

Th17 cells selectively produce IL-17A, IL-17F, IL-21, and IL-22. Among these cytokines, IL-17A and IL-17F have critical roles in various immune responses such as host defense against pathogens and autoimmune and inflammatory conditions [36]. They are members of the IL-17 cytokine family that have high amino acid sequence homology and share a common receptor subunit, IL-17 receptor A (IL-17RA), and IL-17 receptor C (IL-17RC). Since IL-17A and IL-17F can form homo- and heterodimers because of their sequence homology [37], they may have similar functions. These include recruiting and activating neutrophils as well as stimulating other structural cells to release proinflammatory mediators, such as IL-6, IL-1*β*, TNF-*α*, granulocyte macrophage colony-stimulating factor (GM-CSF) and granulocyte colony-stimulating factor (G-CSF), C-C motif (CC) chemokines, C-X-C motif (CXC) chemokines, antimicrobial peptides, and metalloproteinases [36, 38]. However, IL-17A plays important roles in the development of autoimmunity, inflammation and tumors and in host defense against bacterial and fungal infections, whereas IL-17F has a role mainly in mucosal host defense mechanisms [38]. It is therefore likely that the biological role of these cytokines in immune responses might not be identical.

## 3. Th17-Associated Cytokines and Steroid-Insensitive Asthma

### 3.1. Increased Expression of IL-17A in Patients with Asthma

Since some phenotypes of asthma cannot be explained using classical Th1/Th2 theory, numerous recent studies have suggested possible roles of IL-17A and IL-17F in asthma. Since the role of IL-17F in the pathogenesis of asthma is reviewed elsewhere in the same issue (see Ota et al. [39]), we describe the role of IL-17A in asthma. Increased levels of IL-17A (or IL-17) protein and messenger RNA were detected in the sputum [15, 40, 41], bronchoalveolar lavage fluids (BALF) [41], bronchial tissues [16, 42–44], peripheral mononuclear cells (PBMCs) [45, 46], and serum [17, 47, 48] from patients with asthma. It was demonstrated that expression of an IL-17A receptor unit, IL-17RA and IL-17RC [49], ROR*γ*t and RORC, which encodes ROR*γ*t, was also increased in the bronchial tissues and PBMCs of asthmatic patients [45, 46]. The expression levels of IL-17A correlated with airway hyperresponsiveness (AHR) [40] and clinical severity [16, 17, 43, 45, 47, 48], suggesting that IL-17A may contribute to the pathogenesis of a certain type of asthma.

### 3.2. Neutrophilic Airway Inflammation

Several studies have suggested the involvement of neutrophils in severe asthma [1, 9–12]. Since IL-17A is capable of inducing neutrophil influx directly into inflammatory sites or indirectly [36, 38], it is natural to consider a link between IL-17A and airway neutrophilia. In asthmatic patients, the expression levels of IL-17A correlated with the levels of C-X-C motif ligand (CXCL) 8 and neutrophilic airway inflammation [15]. By contrast, the results from another study demonstrated no association between IL-17A expression and neutrophilic inflammation despite an enhanced expression of IL-17A in bronchial submucosa of asthmatics [42]. However, a relationship between IL-17A expression and airflow limitation and sputum neutrophil counts were shown suggesting a potential role for IL-17A in a neutrophilic type of asthma [42].

These clinical observations were extended and confirmed in experimental animal models. To examine whether IL-17 can induce neutrophil recruitment, recombinant IL-17 was directly administered into rodent airways, which resulted in increased numbers of neutrophils and CXC chemokine expression [50–52]. In allergic animal models, neutrophilic airway inflammation was induced by ovalbumin (OVA) exposure in two strains of OVA-specific T cell receptor (TCR) expressing transgenic mice, OTII and DO11.10 [53, 54], and was found to be attenuated in genetic mice lacking IL-17 [54]. Similar results were observed using Th17-polarized cells obtained from DO11.10 mice. Adoptive transfer of those cells into severe combined immunodeficiency (SCID) or naïve BALB/c mice induced the development of airway neutrophilia and AHR accompanied by increased expression of IL-17 and neutrophil chemoattractants such as CXCL 5, CXCL8, and G-CSF, after OVA challenge. These responses were abolished by depleting IL-17A using antibodies and in genetic mice lacking IL-17 receptor (IL-17R) [50, 55]. Lajoie et al. recently reported interesting data using two strains of mice, one with high susceptibility to develop allergen-induced AHR and the other with low susceptibility, each corresponding to severe and mild asthma, respectively. The susceptible strain manifested increased production of IL-17A and Th2 cytokines and severe AHR, which was attenuated by neutralizing IL-17A. In contrast, less susceptible strain exhibited a predominant Th2 cytokine profile and less severe AHR, which was aggravated by IL-17A administration [56]. These studies support the importance of IL-17A as a key regulator in generating neutrophilic inflammation and enhancing the severity of allergen-induced airway responses.

Understanding the underlying mechanisms that cause airway immune responses and promote Th17 polarization has been of great interest. We recently reported that the balance between two types of allergen-induced inflammation, neutrophilic and eosinophilic, is controlled by ROR*γ*t and GATA-3, Th17 and Th2 master transcription factors, respectively. ROR*γ*t-transgenic mice, which overexpress IL-17A, showed enhanced airway neutrophilia and AHR with increased expression of neutrophil chemoattractants in response to allergen exposure, whereas GATA-3-transgenic mice, which overexpress Th2 cytokines, developed enhanced airway eosinophilia and AHR [57]. Similarly, as described above, the transfer of *in vitro* polarized Th17 cells resulted in allergen-induced airway neutrophilia, while that of polarized Th2 cells resulted in allergen-induced AHR accompanied by airway eosinophilia [55]. Furthermore, the route of antigen sensitization, through the skin [58] or airway [59], but not peritoneum, and the duration of antigen exposure, longer rather than short [14, 60, 61], were important for the elicitation of Th17 responses in airway inflammation. These results suggest that the phenotypes of allergen-induced airway inflammation can be determined through the Th2/Th17 balance by both endogenous and exogenous factors.

### 3.3. Contribution of IL-17A to Steroid Insensitivity

There is no doubt that the presence of neutrophils in the airways is one explanation for steroid resistance in asthma. Neutrophilic inflammation tends to respond poorly to steroid therapy as steroids induce apoptosis in eosinophils but increase neutrophil release from the bone marrow, reduce egress of neutrophils from the circulating pool into the marginating pool, and prevent neutrophil apoptosis [62, 63]. Airway neutrophilia and IL-17A and CXCL8 expression were in fact not attenuated by steroid treatment in asthmatics [15]. Likewise, steroids had no effect on increased airway-infiltrating neutrophils and CXC chemokines in Th17-polarized mice following allergen exposure [55, 57] although some studies reported contrary results [14, 43, 51]. Interesting results have been obtained using primary epithelial cells from healthy subjects and asthmatics. Steroid treatment caused a significant reduction in IL-17A-induced IL-6 expression in epithelial cells obtained from normal controls but not in those from asthmatics [44]. Similarly, *in vitro* studies demonstrated that IL-17A-induced CXCL8 production in the bronchial epithelial cell line was normally sensitive to steroids. However, following pretreatment with IL-17A, TNF-*α*-induced CXCL8 production became insensitive [64]. An increase in glucocorticoid receptor (GR)-*β* expression [44] and reduction of histone deacetylase (HDAC) 2 activity [64] in target epithelial cells was suggested as a possible molecular mechanism for IL-17A-induced steroid insensitivity.

### 3.4. Airway Remodeling

Airway remodeling, such as subepithelial collagen deposition and increased airway muscle mass, together with excessive mucus secretion, is important components leading to irreversible air flow limitation that is insensitive to steroid treatment [1]. Several studies demonstrated that modifying the expression of IL-17A did not alter the degree of airway remodeling in an experimental mouse model of asthma [56, 57]. However, sensitized mice with prolonged allergen exposure developed airway remodeling, and its severity positively correlated with the number of CD4^+^IL-17^+^ cells and IL-17 concentration in the airways [61]. Similar results were also observed in Th17-transferred mice [61] and IL-17 transgenic mice [19]. These outcomes were supported by *in vitro* cultures of airway structural cells. IL-17 was shown to promote migration, proliferation, and reduction of apoptosis in smooth muscle cells [65, 66], to stimulate the expression of mucin genes in epithelial cells [67], and to potentiate the production of profibrotic cytokines in fibroblasts and eosinophils [41, 68]. Although it is accepted that T cells, and particularly Th2 cells, play a critical role in the development of asthmatic airway remodeling, these results indicate a possibility that Th17 cells may also contribute to its pathogenesis.

### 3.5. Cross-Regulation between IL-17A and Th2 Responses

A reciprocal negative regulation between Th17 cells and Th2 cells has been considered to exist during immune responses [18, 19, 69]. Th17 cell differentiation is negatively controlled by Th2 cytokines [18, 19]. From another viewpoint, IL-17 administration reduced Th2-type responses, such as airway eosinophil recruitment, AHR, and expression of C-C motif chemokine ligand (CCL) 11 and CCL17, in allergen-exposed mice. Production of Th2 cytokines was also decreased by IL-17 in mediastinal lymph nodes from allergen-exposed mice [70]. In addition, treatment with anti-IL-17 neutralizing antibody enhanced bronchial eosinophil influx and IL-5 expression in an allergen-induced model of asthma [14, 70]. Conversely, CCL11 expression was increased in the lung tissues of IL-17 transgenic mice [19] and in IL-17A-treated airway smooth muscle cells in other experiments [71, 72]. Airway eosinophil recruitment was decreased in IL-17R deficient mice following allergen exposure [70]. These results indicate that, in contrast to exogenous IL-17, endogenous IL-17 is necessary for developing eosinophilic inflammation by inducing CCL11.

Consequently, it is difficult to describe a uniform contribution of IL-17 to the pathogenesis of asthma. It might not be appropriate to have a simple view that Th17 and Th2 cytokines reciprocally counteract each other. In addition, it would also be too straightforward to consider Th17-cell-mediated neutrophilic type of airway inflammation as severe asthma and Th2-cell-mediated eosinophilic type of inflammation as mild asthma. Indeed, Wenzel et al. reported that, when they classified asthmatics with inflammatory cell types in their sputum, patients with increased numbers of both neutrophils and eosinophils presented the most severe clinical features even when treated with steroids [73]. Sputum analysis also confirmed that patients with a simultaneous increase in IL-5 and IL-17A had significantly worse lung function parameters and that uncontrolled asthmatics tended to have higher IL-5 and IL-17A mRNA levels than controlled asthmatics [74]. Consistent with these clinical studies, sensitized mice, cochallenged with allergen and IL-17A, presented increased airway-infiltrating eosinophils and neutrophils and severe AHR, whereas those, challenged with allergen alone, presented only increased airway-infiltrating eosinophils and no AHR, and sensitized mice challenged with IL-17A alone developed moderate airway neutrophilia but not AHR [59]. In another animal study, asthma-susceptible mice were intratracheally exposed to IL-17A, IL-13, or a combination of both to elucidate the cross-regulation between IL-17A and Th2 responses during the development of airway inflammation. AHR was induced by treatment with IL-13, but not by IL-17A alone; however, a significant increase in AHR was demonstrated in mice treated with both IL-13 and IL-17A [56]. Taken together, the production of IL-17A may not simply suppress Th2 responses, but also enhance them to promote a severe phenotype of asthma in certain situations.

## 4. Therapeutic Considerations for Targeting the Th17 Signaling Pathway in Steroid-Insensitive Asthma

**Figure 1:**
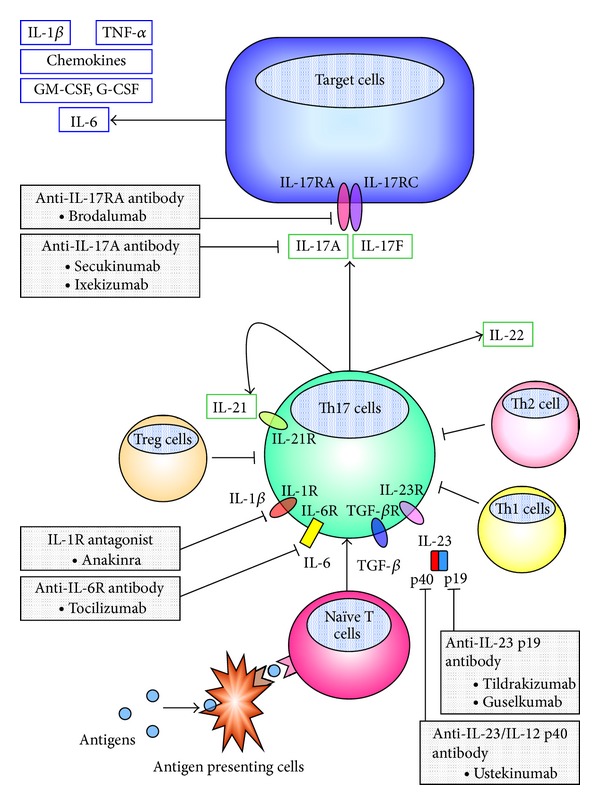
Targeting the Th17 pathway. Helper T (Th) 17 cells are derived from naïve CD4^+^ T cells under the control of transforming growth factor (TGF)-*β*, interleukin (IL)-6, and IL-23 during stimulation by cognate antigen. These cytokines also stimulate Th 17 cells to produce IL-21, which affects Th17 cells themselves to activate a specific transcription factor, ROR*γ*t through autocrine regulation. Other proinflammatory cytokines, IL-1*β* and tumor necrosis factor (TNF)-*α*, may also promote Th17 development. ROR*γ*t regulates both Th17 cell differentiation and production of Th17-signature cytokines, IL-17A, IL-17F, IL-21, and IL-22. Among these cytokines, IL-17A and IL-17F play pivotal roles in the pathogenesis of asthma and share a common receptor subunit, IL-17 receptor A (IL-17RA), and IL-17 receptor C (IL-17RC). Several inhibitors of Th17 pathway are currently under clinical investigation.G-CSF, granulocyte colony-stimulating factor; GM-CSF, granulocyte macrophage colony-stimulating factor; IL-1R, IL-1 receptor; IL-6R, IL-6 receptor, Treg, regulatory T.

Steroids are potent immunosuppressive and anti-inflammatory agents, offering a pivotal role among currently available treatment options for asthma. However, they may function in a nonspecific manner. Since an effective approach to control steroid-insensitive asthma has not yet been developed, novel therapeutic agents that target specific molecular events are required. As some phenotypes of severe asthma are associated with excessive Th17 responses, adjusting Th17 signaling might offer effective therapeutic options for steroid-insensitive asthma. Considering the process of Th17 cell differentiation and function there are a number of theoretical candidate therapeutic targets. These include Th17-cell differentiation factors IL-6, IL-1*β*, and IL-23, Th17 cytokine IL-17A, Th17-cell specific transcription factor ROR*γ*t, IL-17A/F receptor IL-17RA, and Th17-cell downstream inflammatory mediators (Figure 1). However, since Th17 immune responses are also important for host defense and possibly antitumor immunity, their favorable roles, as well as pathogenic roles, should be considered when inhibiting Th17 signaling pathways in the clinic. Among these targets, several are currently being tested in clinical trials as described below.

### 4.1. Blocking IL-17A and IL-17RA

Based on results obtained from cellular, animal, and human studies, clinical studies in autoimmune and inflammatory diseases, such as psoriasis, rheumatoid arthritis, ankylosing spondylitis, uveitis, and Crohn's disease, have already been conducted to confirm anti-inflammatory effects by blocking IL-17A [75]. Compared with nonspecific immunosuppressive agents, targeting IL-17A is considered advantageous in only attenuating inflammation but not host defense, because IL-17F may have the potential to compensate for immunocompromised conditions [38].

Currently, numerous clinical studies examining the effectiveness of monoclonal antibodies against IL-17A are underway [75]. Phase II trials of secukinumab and ixekizumab demonstrated positive results for the substantial relief of symptoms, with satisfactory safety, in patients with Th17-related diseases, such as plaque psoriasis, psoriatic arthritis, rheumatoid arthritis, uveitis, and ankylosing spondylitis, but not those with Crohn's disease [76–82]. Brodalumab, a monoclonal antibody against IL-17RA, demonstrated favorable results as well in patients with plaque psoriasis [83, 84]. Equivocal findings were reported in the clinical trial of brodalumab in patients with moderate-to-severe asthma [85]. They showed benefits of brodalumab in improving clinical symptoms in the high bronchodilator reversibility subgroup, despite failing to achieve clinical or statistical improvements in the overall subjects with asthma. In addition, phase II trials of secukinumab for uncontrolled asthma have since been initiated, in which favorable results are expected (NCT01478360).

### 4.2. Blocking IL-23 and IL-1*β*

As IL-23 is crucial to the development of Th17 cells, a monoclonal antibody against the p40 subunit of IL-23/IL-12, ustekinumab, has also been investigated in clinical trials for the treatment of immune-mediated diseases such as psoriatic arthritis and Crohn's disease [86, 87]. Its efficacy, safety, and tolerability in the management of psoriasis are generally accepted; therefore, it is already approved to treat moderate-to-severe plaque psoriasis in several countries. Clinical trials using a monoclonal antibody against the p19 subunit of IL-23, expected to specifically inhibit IL-23, are underway to evaluate its efficacy in patients with plaque psoriasis and rheumatoid arthritis [75].

IL-1*β* may also be a therapeutic target because it has an important role in the development of Th17 responses. Anakinra, an IL-1 receptor (IL-1R) antagonist is already available for clinical use for rheumatoid arthritis and is now being investigated to determine whether to reduce endotoxin-induced airway inflammation (NCT01369017). Since endotoxin is believed to be associated with asthma exacerbations, anakinra may have a possibility to be one of the therapeutic options for asthma.

Although clinical trials of agents that block IL-23 or IL-1*β* have not been conducted in patients with asthma, future studies are required to elucidate the potential of treatments targeting IL-23 or IL-1*β* as novel therapeutic strategies for steroid-insensitive asthma.

### 4.3. Blocking IL-6

Recent studies showed that IL-6 is important for promoting Th17 cell differentiation and orchestrating downstream pathways of Th17 immune responses to cause inflammatory and autoimmune disorders such as rheumatoid arthritis and multiple sclerosis [23, 88, 89]. IL-17A also contributes to autoimmunity by triggering a positive feedback loop via IL-6 induction [23]. IL-17A-biased immune conditions may accelerate IL-6 production and in turn, an excessive amount of IL-6 may amplify upstream of Th17 immune responses to promote Th17-driven inflammation. Therefore, blocking this amplification loop might be important for the resolution of inflammation. In asthmatics, the expression of IL-6 and soluble IL-6 receptor (IL-6R) were increased in the serum and airways [90–92]. In addition, a novel variant of IL-6R was recently identified in a genome-wide study and was significantly associated with asthma risk [93]. In an animal study of allergic airway inflammation, we demonstrated that IL-6 production was increased in IL-17-induced steroid-insensitive airway inflammation and that both airway neutrophilia and AHR were effectively attenuated by treatment with an anti-IL-6R antibody [57]. These results indicated that IL-6, as well as IL-17, is a potential target for the treatment of Th17-driven steroid-insensitive airway inflammation.

Inhibition of the Th17 pathway by IL-6 blockade has recently been proposed as a treatment option in various autoimmune and inflammatory diseases [94]. A humanized anti-IL-6R antibody, tocilizumab, which was demonstrated to be therapeutically effective for rheumatoid arthritis, systemic juvenile idiopathic arthritis, and Castleman's disease, is already available for clinical use [88]. Consequently, it can be expected to become a novel therapeutic option for steroid-insensitive asthma, too.

## 5. Conclusions

Our understanding of the immunologic cascade in the pathogenesis of asthma has greatly advanced over the last 20 years, as various Th cell subsets have been identified. Numerous studies have been performed to determine the precise mechanism of treatment-insensitive severe asthma and to identify targets that may provide therapeutic benefits. From these studies, excessive Th17 responses have been shown to be key factors involved in steroid-insensitive asthma. In this review, we discussed that Th17-associated cytokines might be potential targets to alter excessive Th17 signaling and might offer advantages over classic therapies, such as steroids, for patients with severe asthma. Several clinical trials targeting Th17 cytokines have been initiated, and we should continue to focus on this issue to improve the outcomes of uncontrolled asthma that is resistant to conventional therapies.

## Abbreviations

AHR: Airway hyperresponsiveness
BALF: Bronchoalveolar lavage fluids
CC: C-C motif
CCL: C-C motif chemokine ligand
CXC: C-X-C motif
CXCL: C-X-C motif ligand
G-CSF: Granulocyte colony-stimulating factor
GM-CSF: Granulocyte macrophage colony-stimulating factor
GR: Glucocorticoid receptor
HDAC: Histone deacetylase
IFN: Interferon
IL: Interleukin
IL-17RA: IL-17 receptor A
IL-17RC: IL-17 receptor C
IL-1R: IL-1 receptor
IL-6R: IL-6 receptor
LTi: Lymphoid tissue inducer
NK: Natural killer
NKT: Natural killer T
OVA: Ovalbumin
PBMCs: Peripheral mononuclear cells (PBMCs)
ROR*γ*t: Retinoic acid-related orphan receptor-*γ*t
SCID: Severe combined immunodeficiency
STAT3: Signaling transducer and activator of transcription 3
TCR: T cell receptor
TGF: Transforming growth factor
Th: Helper T
TNF: Tumor necrosis factor
Treg: Regulatory T.
